# Conformal 3D Printing Algorithm for Surfaces and Its In Situ Repair Applications

**DOI:** 10.3390/mi15070920

**Published:** 2024-07-17

**Authors:** Jundong Tang, Yongli Dong, Lixiang Cai, Qian Zhu, Jianping Shi

**Affiliations:** 1 School of Electrical and Automation Engineering, Nanjing Normal University, Nanjing 210023, China; 27210233@njnu.edu.cn (J.T.); d17682766829@163.com (Y.D.); 18306298121@163.com (L.C.); 13140793018@163.com (Q.Z.); 2 Jiangsu Key Laboratory of 3D Printing Equipment and Manufacturing, NARI School of Electrical and Automation Engineering, Nanjing Normal University, Nanjing 210023, China

**Keywords:** conformal 3D printing, in situ printing, bioprinting, tissue repair

## Abstract

Conformal 3D printing can construct specific three-dimensional structures on the free-form surfaces of target objects, achieving in situ additive manufacturing and repair, making it one of the cutting-edge technologies in the current field of 3D printing. To further improve the repair efficacy in tissue engineering, this study proposes a conformal path planning algorithm for in situ printing in specific areas of the target object. By designing the conformal 3D printing algorithm and utilizing vector projection and other methods, coordinate transformation of the printing trajectory was achieved. The algorithm was validated, showing good adherence of the printing material to the target surface. In situ repair experiments were also conducted on human hands and pig tibia defect models, verifying the feasibility of this method and laying a foundation for further research in personalized medicine and tissue repair.

## 1. Introduction

Traditional 3D printing primarily operates on a planar surface, constructing objects layer by layer from the bottom up, with each layer maintaining the same Z coordinate during printing [1]. In contrast, the conformal 3D printing tool head moves simultaneously along the XYZ axes, enabling the creation of specific three-dimensional structures on free-form surfaces [2,3]. This approach eliminates spatial limitations in printing, thereby expanding the range of 3D printing applications [4,5]. Additionally, direct printing on non-planar surfaces offers advantages over traditional 3D printing in terms of material cost and structural strength [6]. Currently, this technology is mainly utilized in the printing of conformal antennas and electronic devices both domestically and internationally. Compared to traditional circuits, curved conformal circuits occupy less space and enable the miniaturization of electronic devices [7,8,9,10].

In situ 3D printing is an emerging tissue engineering technology tailored to meet the specific needs of patients. It utilizes prepared tissue-specific bioinks to print grafts directly at the site of biological defects, promoting tissue regeneration [11] and effectively reducing risks such as infection [12,13]. The printed structures also exhibit high mechanical properties [14,15]. In vitro bioprinted structures must be cultured and induced with a lower degree of functionalization. In contrast, in situ printing can leverage the more complex and comprehensive in vivo stimulation environment to facilitate deep functionalization of bioprinted structures [16]. In situ 3D bioprinting primarily involves two approaches: robotic-assisted 3D bioprinting [17] and handheld 3D bioprinting [18,19]. There have been cases of skin [20] and bone [21] restoration using in situ 3D printing, but none of them require much in the way of flat patterns or structures to be printed, which can be solved by conformal 3D printing. Applying conformal 3D printing to in situ printing can enhance the adhesion between the printing material and the object surface, allowing for the creation of specialized structures along specified trajectories and reducing postoperative healing time.

The skin is the largest organ of the human body, and its trauma repair is a representative case of dermal tissue repair. When large areas of skin are damaged, autologous skin donors cannot be used, and repair can only be achieved through skin transplantation, which is often limited by immune rejection [22]. The advent of 3D printing technology has made artificial skin a possibility, with current treatments often involving in vitro printing and transplantation [23]. In situ 3D printing not only allows for the repair of large areas of traumatized skin [24] but also reduces the problem of immune rejection by using stem cells, such as iPSCs, as the printing material [25,26]. Traditional in situ skin printing mainly focuses on filling wounds, where the wound morphology is scanned, and the printing material is deposited at specified locations, lacking structural selectivity [24]. Filling materials used in traditional methods have low strength and can easily collapse under their weight, causing the material to fall off [27].

Bone defects are common clinical injuries. Although bones have strong self-healing capabilities, the probability of self-healing decreases significantly when the damage exceeds a critical size [28]. Common clinical repair methods include induced membrane technology, distraction osteogenesis, autologous/allogeneic transplantation, and vascularized fibula transplantation [29]. However, these methods are often plagued by issues such as susceptibility to infection, slow recovery, low durability, and insufficient autologous bone grafts [30]. In situ 3D printing of biological scaffolds can concentrate cells at the damage site and guide cell proliferation [31]. The printed scaffolds not only meet strength requirements but can also be adjusted to create complex spatial structures with varying pore sizes [32]. While this method promotes growth in the defective area, it is challenging to use different materials to separately repair bone marrow and the exoskeleton when both are injured, leading to a repaired part that differs significantly in appearance from the original.

In response to these challenges, this study explores the design of conformal 3D printing algorithms and the selection of printing paths to achieve conformal 3D printing at the damaged site, thereby completing the repair task. This provides a reference for the practical application of this technology in clinical medicine.

## 2. Materials and Methods

### 2.1. Multi-Nozzle 3D Printing System

**Figure 1:**
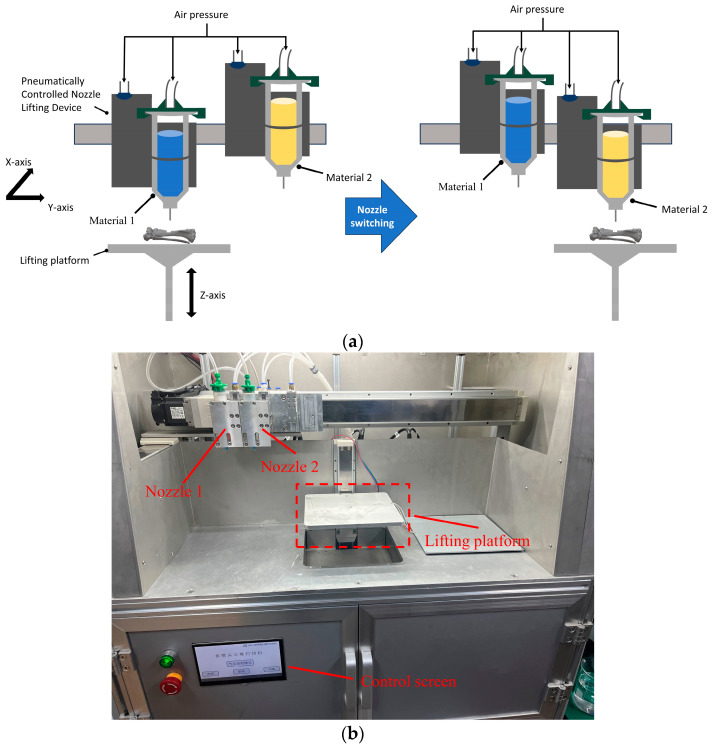
Multi-nozzle 3D printing system. (**a**) Working diagram; (**b**) physical picture.

This study utilized a custom-built multi-nozzle 3D printing system for the experiments [33]. The system is driven by a Duet 2 WIFI control board, which supports four printing nozzles and manages nozzle switching via air pressure. Channels 1 to 3 are configured as air pressure extrusion nozzles by default. The biological material in the syringe is extruded through controlled air pressure, with the parameters adjustable via a touchscreen interface. Figure 1 offers a comprehensive visualization of both the schematic and the actual setup of this state-of-the-art multi-nozzle 3D printing system, providing valuable insights into its intricate workings and capabilities. Through the utilization of this advanced printing technology, this study aims to push the boundaries of additive manufacturing and pave the way for novel applications in various fields.

### 2.2. Preparation of Printing Materials

Hydrogels are composed of a hydrophilic, branched 3D polymer network similar to the natural extracellular matrix (ECM) in the body, and their biocompatibility makes cell-biomaterial interactions superior to synthetic polymers, making them suitable for tissue regeneration applications [27,34]. Additionally, the hydrogel network can promote matrix remodeling, cell migration, and cell adhesion in a three-dimensional environment, all of which are essential for the normal development of functional tissues [35]. Therefore, hydrogel was utilized as the material in this study to simulate actual printing conditions.

The experimental materials were prepared as follows: Pluronic F-127 (Sigma-Aldrich, Saint Louis, MO, USA) was dissolved in distilled water at a concentration of 40% mass volume (*w*/*v*). After allowing it to stand for 30 min at 45 °C until fully dissolved, the mixture was stirred evenly. Next, the prepared hydrogel was divided into two equal portions. To showcase the multi-material printing results, one portion was enriched with a single drop of red food coloring and meticulously mixed.

### 2.3. Preparation of Hand and Bone Defect Models

**Figure 2:**
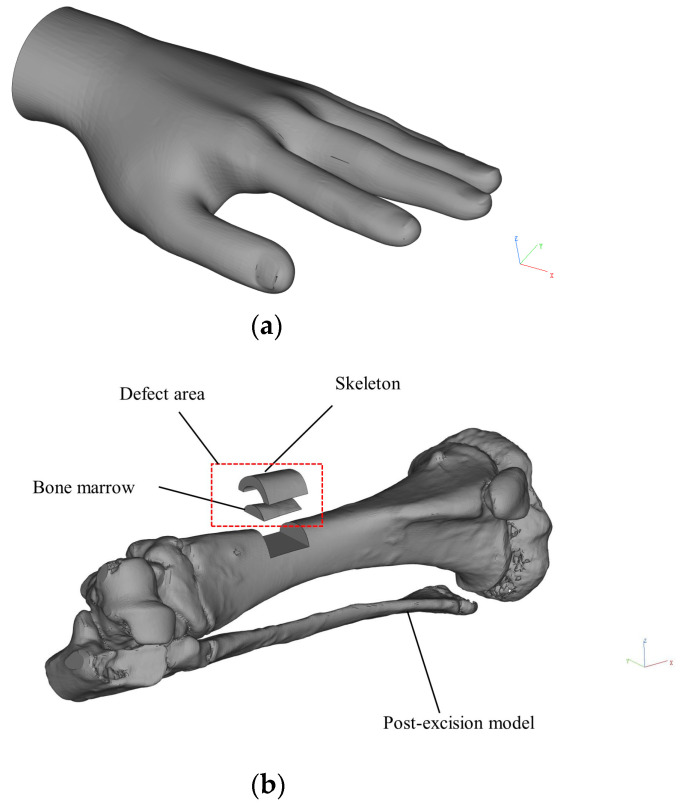
The models used:(**a**) human hand model; (**b**) pig tibia model.

The human hand model was sourced from the online 3D model-sharing platform Diwei Model (www.3dwhere.com (accessed on 14 July 2024)), as illustrated in Figure 2a, and printed using PLA material. To streamline the subsequent printing experiments, the model’s support structure remained intact after printing.

In situ, 3D printing is commonly employed for skin and bone repair. To validate the versatility of conformal 3D printing for in situ repair, this study utilized pig tibia models to simulate bone repair experiments. A scan was performed on the tibia of the experimental Bama pig, and the resulting digital model was converted to the STL file format. This file underwent editing in Magics 21.0 software (Materialise, Leuven, Belgium). A 12 mm long, 14 mm wide, and approximately 6 mm deep section was manually removed from the bone model to simulate the defect. Subsequently, the bone marrow model was generated through a Boolean operation on the defect part and the resected pig tibia model, as illustrated in Figure 2b. The STL files of the resected model, defective part model, and bone marrow model were saved individually. Using PLA material, the resected pig tibia model was printed without removing the model support.

## 3. Conformal 3D Printing Algorithm

### 3.1. Basic Idea of Algorithm

Conformal 3D printing enhances traditional flat 3D printing by modifying the XYZ coordinates of the printing motion trajectory. This technique projects the planar trajectory onto the surface of the printing substrate, enabling effective coordinate transformation [36,37,38].

**Figure 3:**
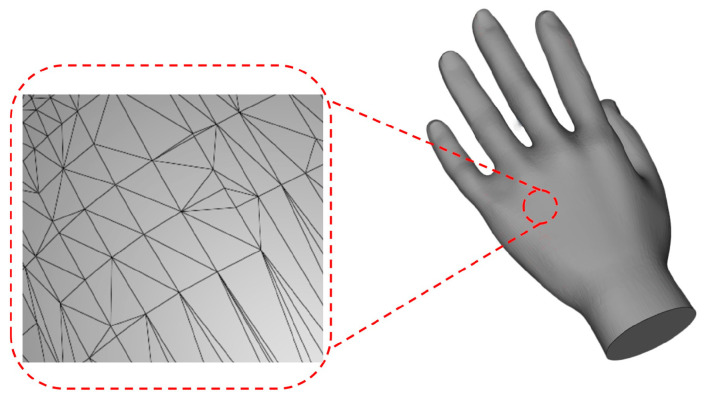
Triangular meshes on the surface of the human hand model.

The algorithm requires the target trajectory and the STL file of the printing matrix as inputs. The target trajectory is a sequence of points on a two-dimensional plane, provided in XY coordinates. As depicted in Figure 3, the STL file represents the three-dimensional object surface using triangular meshes. This file includes crucial information, such as the normal vectors of all triangular meshes and the spatial coordinates of their three vertices [39,40].

**Figure 4:**
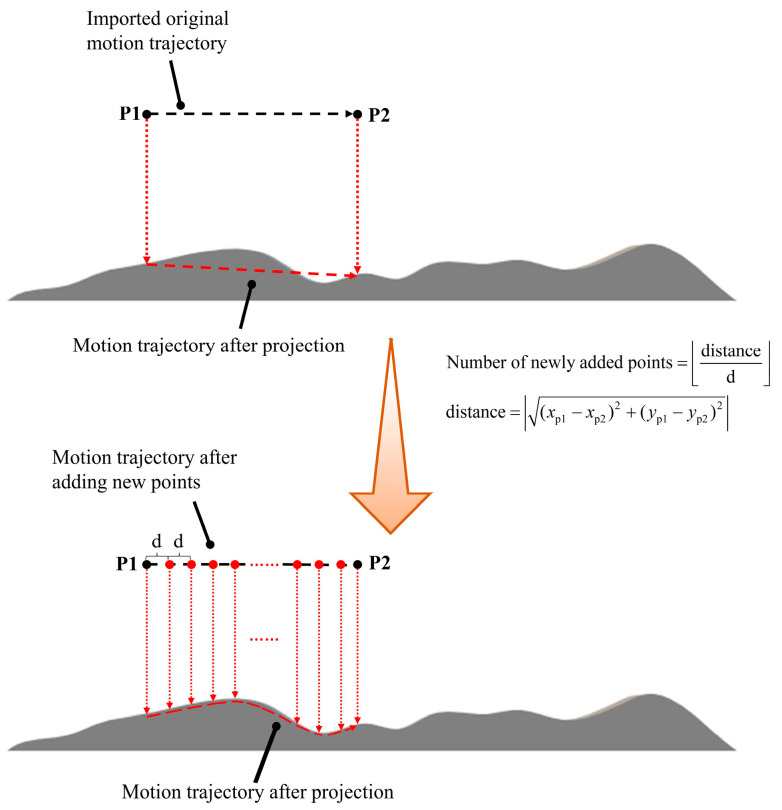
The change process of the motion trajectory before and after adding new points.

The horizontal distance between trajectory points generated using CAD2020 software may be too large, posing challenges for conformal printing on a non-planar substrate. When two points with an excessively large horizontal distance are projected onto the surface, this can prevent conformal printing and may even cause collisions with the printing nozzles. To mitigate this issue, additional points are inserted between the original trajectory points. The spacing of these new points, denoted as d, can be adjusted to control the conformal accuracy. A smaller d value results in a printing track that more closely follows the surface of the printing substrate. In this study, a spacing of d = 1 mm was selected, as shown in Figure 4.

For the input STL file, the vertex coordinate information for each triangular mesh must be extracted. Based on the coordinates of the three vertices of each triangular mesh, Formulas (1) and (2) are used to derive the plane equation for the triangular mesh.

(1) Ax+By+Cz+D=0

where

(2) $$\left\{ \begin{array}{l} A=(y_{2}-y_{1})(z_{3}-z_{1})-(z_{2}-z_{1})(y_{3}-y_{1})\\ B=(x_{3}-x_{1})(z_{2}-z_{1})-(x_{2}-x_{1})(z_{3}-z_{1})\\ C=(x_{2}-x_{1})(y_{3}-y_{1})-(x_{3}-x_{1})(y_{2}-y_{1})\\ D=-(Ax_{1}+By_{1}+Cz_{1}) \end{array}\right.$$

**Figure 5:**
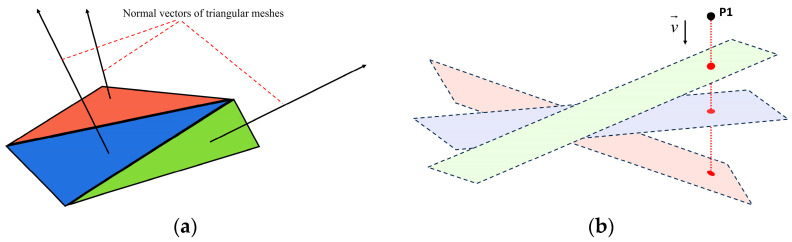
The projection of the trajectory point on the plane where the triangular mesh is located. (**a**) Simple diagram of model triangular mesh; (**b**) single trajectory point projection diagram.

Given the projection vector $v=(v_{x},v_{y},v_{z})$, all newly added points are projected along the vector $\vec{v}$ onto the plane containing the triangular meshes of the printing matrix, as shown in Figure 5. For a single original trajectory point P(x,y,z), Formula (3) is used to calculate its projection point on an individual triangular mesh plane.

(3) $$\left\{ \begin{array}{l} x_{\mathrm{project}}=x+v_{x}t\\ y_{\mathrm{project}}=y+v_{y}t\\ z_{\mathrm{project}}=z+v_{z}t\\ t=-\frac{\left(Ax+By+Cz+D\right)}{\left(Av_{x}+Bv_{y}+Cv_{z}\right)} \end{array}\right.$$

From this, the projection point $P_{\mathrm{project}}(x_{\mathrm{project}},y_{\mathrm{project}},z_{\mathrm{project}})$ of a single original trajectory point on a single triangular mesh plane is obtained. By traversing all triangular mesh planes, all projection points of this point can be calculated. In this study, the projection vector *v* = (0, 0, −1) is selected, so the obtained projection point has the same XY coordinates as the trajectory point, and only the Z coordinate is transformed. Although the trajectory point has a projection point on a triangular mesh plane, the projection point of this point is not necessarily within the triangular mesh range of this plane. Formula (4) is used to filter out projection points that are not within the range of the triangular mesh. If m and n in the formula exist and satisfy the condition: m≥0,n≥0,m+n≤1, it means that this point is within the range of the triangular mesh. Due to the overlap of triangular meshes on the model surface, multiple projection points persist after filtering. It becomes essential to choose the projection point with the shortest spatial distance from the trajectory point P among the remaining projection points as the final projection point.

(4) $$P_{\mathrm{project}}=(1-m-n)\cdot T_{1}+m\cdot T_{2}+n\cdot T_{3}$$

**Figure 6:**
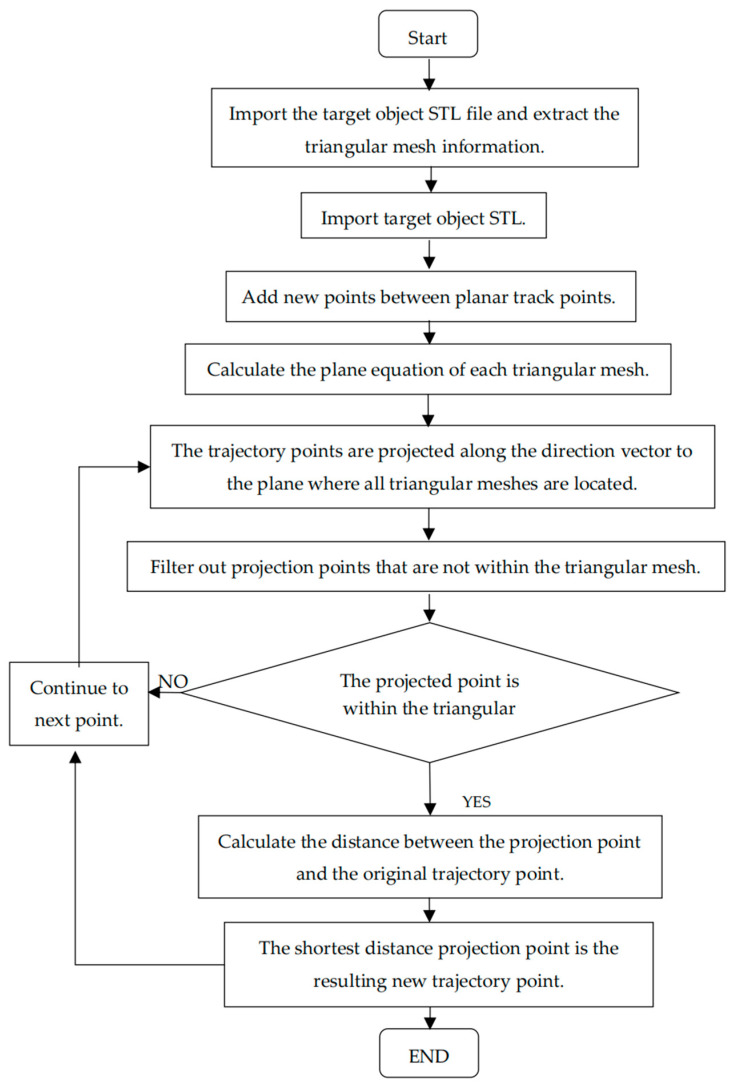
Conformal 3D printing algorithm flow chart.

Employing the described method to traverse and compute all trajectory points, the resulting coordinates of all final projection points can be seamlessly outputted as Gcode, defining the precise motion path of the printing nozzle. Figure 6 provides a visual representation of the conformal 3D printing algorithm flowchart.

In printing processes such as FDM, that demand filament extrusion volume, recalibration becomes imperative due to the non-linear trajectory. This entails recalculating the total extrusion amount, which can be achieved using the Formula (5).

(5) $$\left\{ \begin{array}{l} e(i)=k\cdot [l+\sum e(i-1)]\\ l=\sqrt{{\left(x_{i}-x_{i-1}\right)}^{2}+{\left(y_{i}-y_{i-1}\right)}^{2}+{\left(z_{i}-z_{i-1}\right)}^{2}}\\ k=\frac{D}{W} \end{array}\right.$$

where e(i) is the extrusion amount advancing to the ith point, l is the spatial distance between the i-1th point and the ith point, D is the nozzle diameter, and W is the wire diameter.

### 3.2. Algorithm Verification

This experiment employed Python 11.0 to implement the algorithm, utilizing numerical calculation libraries such as NumPy (V1.24.0), Pandas (V2.2.0), and math, alongside the stl (V0.0.3) library for processing the input STL file.

A free-form surface model was crafted in SOLIDWORKS 2018 (Dassault Systèmes, Boston, MA, USA) modeling software to act as the printing matrix, with PLA utilized as the printing material. Ensuring the precise processing of each point by the algorithm necessitates generating a trajectory covering the entire upper surface of the printing substrate. This was achieved by slicing the printing substrate STL file into a Gcode file using Simplify3D (V4.0.1).

To validate the trajectory coverage, the trajectory code of the first printing layer in the Gcode file was extracted as the target trajectory. Subsequently, trajectory coordinate information was gleaned using Python regular expressions.

**Figure 7:**
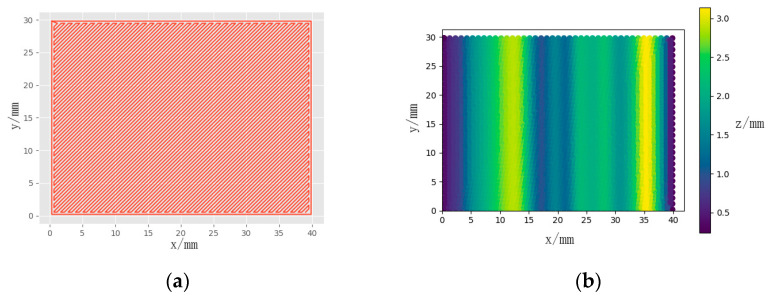
Algorithm processing of printing trajectories. (**a**) Algorithm input trajectory; (**b**) algorithm output trajectory.

By providing the printing matrix STL file and trajectory as inputs, the algorithm generated the conformal 3D printing motion trajectory. Figure 7a depicts the plane trajectory input into the algorithm, while Figure 7b illustrates the curved conformal printing trajectory produced by the algorithm. The color gradient indicates z-direction values, with lighter hues representing larger values and darker tones signifying proximity to 0 mm.

**Figure 8:**
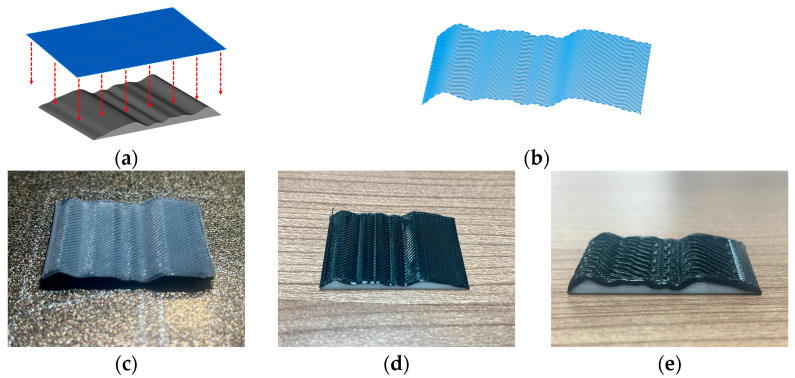
Conformal 3D printing verification experiment. (**a**) Plane trajectory and printing matrix; (**b**) Conformal 3D printing trajectory; (**c**) Single layer PLA printing result; (**d**) Single-layer TPU printing results; (**e**) Multi-layer TPU printing results.

Conformal 3D printing was performed using the Bambu Lab A1 mini 3D printer (Bambu Lab, Shenzhen, China) with a nozzle diameter of 0.4 mm. Two types of filaments, PLA (white, 1.75 mm) and TPU (black, 1.75 mm, 95A), were chosen for printing. The layer height was set to 0.2 mm, with printing speeds of 1200 mm/min for PLA and 400 mm/min for TPU. Figure 8 showcases the printing outcomes, including a single layer of white PLA on a black printing substrate, as well as the results of single and multi-layer printing of black TPU on a white printing substrate.

The experimental outcomes incontrovertibly validate the algorithm’s adeptness in precisely projecting every trajectory point, thus seamlessly achieving conformal 3D printing on the complex free-form surface of the target object.

## 4. In Situ Conformal Printing

### 4.1. Conformal Printing of Human Hand Surface

The skin on the hand, frequently exposed to the external environment, is prone to damage. In this experiment, conformal 3D printing was employed on the surface of a human hand model to simulate the process of skin repair.

**Figure 9:**
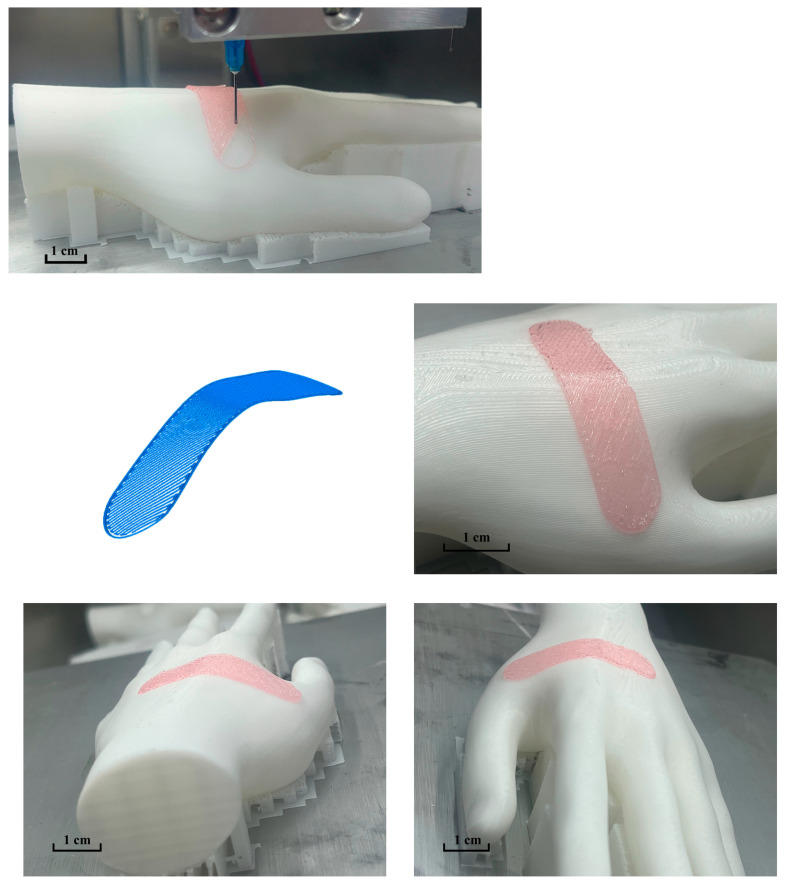
Conformal printing trajectory and printing effect on human hand surface.

The wound repair model intended for printing and application was designed using SOLIDWORKS 2018 (Dassault Systèmes, USA) software. The model created for this experiment measured 40 mm in length and 10 mm in width. Importing this model’s STL file into Simplify3D (Simplify3D, Cincinnati, OH, USA), it was positioned at the coordinate origin and sliced. The nozzle motion trajectories x, y, and z in the resulting Gcode file were selected as the input trajectories for the algorithm. The generated conformal 3D printing trajectory was then uploaded to the multi-nozzle 3D printing system. The nozzle diameter was set at 0.39 mm, with extrusion air pressure adjusted to 260 kPa and a printing speed of 400 mm/min. To prevent collisions between the nozzle and the printing substrate, a 0.2 mm height was reserved during *Z*-axis calibration. Figure 9 illustrates the conformal printing effect on the surface of the human hand.

The printing results reveal the successful application of printing material onto the surface of the human hand model, with no wire breakage observed during the process. Additionally, the printed shapes exhibit remarkable fidelity, effectively fulfilling the intended repair task.

### 4.2. Simulated Repair and Printing of Pig Tibia Defects

This experiment employs multiple nozzles to simulate repair printing on both the bone marrow and the bone. First, the bone marrow substitute is printed, followed by switching the nozzles to apply the conformal printing method for the bone substitute onto the printed bone marrow substitute. This enables multi-material repair printing for defective parts, ensuring the final appearance matches the overall bone shape post-printing.

To print the bone marrow substitute, the STL file of the bone marrow model is imported into Simplify3D (Simplify3D, USA) for slicing, with a layer thickness set to 0.4 mm. A nozzle equipped with pigment-free hydrogel is utilized, with a diameter of 0.39 mm, air pressure set to 260 kPa, and a printing speed of 400 mm/min.

**Figure 10:**
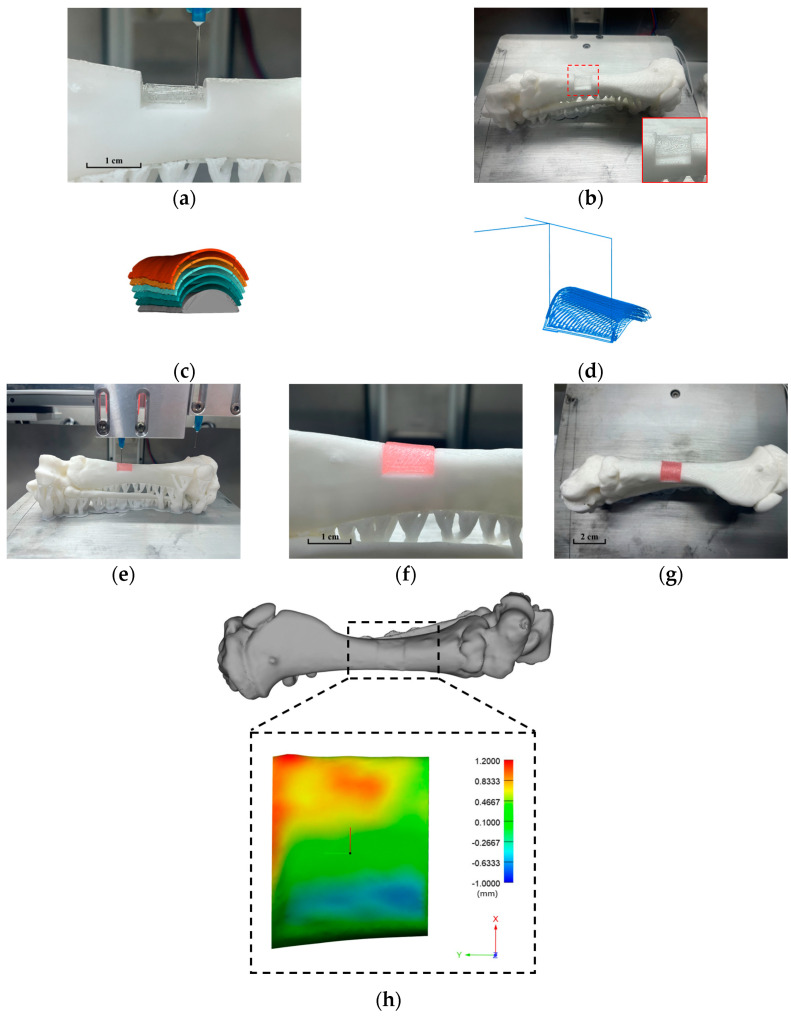
Simulated repair and printing of pig tibia defects: (**a**) bone marrow printing process; (**b**) bone marrow printing effect; (**c**) bone part hierarchical structure diagram; (**d**) Conformal printing trajectory of the bone part; (**e**) bone printing process; (**f**) skeleton printing effect (side view); (**g**) skeleton printing effect (top view); (**h**) three-dimensional comparison of repaired bone and intact bone.

For better restoration of the defective part’s appearance, conformal 3D printing is employed for printing the bone part. The STL file of the defective bone part model is imported into Magics 21.0 (Materialise, Leuven, Belgium) software. An offset is initiated from the upper surface of the outer layer, segmented every 0.4 mm, and processed a total of 5 times. Each processed model represents the ideal printing shape of each layer, with the projection of the lower surface of each model on the horizontal plane selected as the original trajectory for printing and input into the algorithm. Each printing layer is performed on the updated printing matrix model after the previous layer is printed. Printing is conducted using a nozzle equipped with pigmented hydrogel, with a diameter of 0.39 mm, extrusion air pressure set to 311 kPa, and a printing speed of 400 mm/min (Video S1). Figure 10 depicts the simulation printing experiment for repairing the defective part of the pig tibia and printing accuracy analysis.

The printing results clearly demonstrate that both the bone marrow and bone substitutes exhibit a remarkable degree of conformity with the resurrected model in terms of appearance, with no material collapse observed.

To further verify the effectiveness of the repair, an accuracy analysis was conducted on the 3D-printed results for repairing defects in the pig tibia. A 3D scanner was utilized to obtain the 3D data model of the repaired pig tibia, which was then compared with the original pig tibia model. The three-dimensional comparison data is depicted in Figure 10h.

In this representation, green indicates a complete overlap between the two models. As the color transitions from green to red, the three-dimensional error gradually increases, while a shift toward dark blue signifies an increase in the three-dimensional error in the negative direction. The error interval ranges from −0.6527 mm to 1.1947 mm.

Upon analysis, it is observed that the overall printing part is slightly offset in the positive direction of the x-axis. This discrepancy may be attributed to errors in the placement of the target printing substrate. However, the overall printing accuracy remains relatively high.

## 5. Conclusions

A conformal 3D printing algorithm was designed, employing a method that projects plane trajectory points onto the surface of the printing substrate along the direction vector. This process facilitates the conversion from a plane trajectory to a curved surface conformal trajectory. During the algorithm verification phase utilizing the FDM printing process, the printing filament and substrate exhibit excellent compatibility, accommodating different printing filaments.

Experimental validations conducted on human hand and pig tibia models affirm the feasibility of conformal 3D printing technology in repairing free-form surfaces. The experimental segment employs the human hand and defective Bama pig tibia as exemplars to showcase the applicability of conformal 3D printing in in situ repair. For hand surface restoration, a self-constructed multi-nozzle 3D printing system successfully achieves conformal 3D printing, offering a novel approach to skin repair. For the porcine tibia defect repair, the study demonstrates the successful repair of bone defects with relatively high accuracy through in situ 3D printing of bone marrow substitutes, followed by nozzle switching for conformal 3D printing of bone substitutes. These outcomes underscore the technology’s potential applications in orthopedics.

In conclusion, this study underscores the promising performance of conformal 3D printing technology in in situ tissue repair, offering valuable insights for its clinical application in tissue engineering and repair. Due to the limitations in equipment accessibility and the complexity of pre-experimental preparations, this study was confined to simulation experiments without the inclusion of biological experiments. In future research, dual robotic arms will be employed to collaborate in bone repair. The findings from this study will be applied to actual biological experiments to further promote the clinical application of 3D printing technology.

## Data Availability

The data presented in this study are available upon request from the corresponding author.

## Supplementary Materials

The following supporting information can be downloaded at: https://www.mdpi.com/article/10.3390/mi15070920/s1, Video S1: Video of Tibia Repair Printing.
